# Incompletely Theorized Agreement and Accommodated Disagreement in the Surrogate Wars

**DOI:** 10.1007/s10730-025-09563-8

**Published:** 2025-10-15

**Authors:** Takunda Matose

**Affiliations:** Cincinnati Children’s Hospital Medical Center, University of Cincinnati College of Medicine, 3333 Burnet Ave, Cincinnati, OH 45229 USA

**Keywords:** End-of-life, Incompletely theorized agreements, Surrogate decision-making, Deep disagreement

## Abstract

In this commentary, I argue that while the value-systems of healthcare providers and surrogate decision-makers might appear to be incommensurable, they share enough common ground over high-level principles that meaningful engagement between the two groups is possible. This engagement can happen through incompletely theorized agreements that allow discussants to focus on shared values while adhering to competing value-systems. Nonetheless, I argue that the privileging of both healthcare providers and their best interest value system within healthcare creates a continued and unjustified subordination of surrogate decision-makers that threatens to undermine the possibility of meaningful engagement between the two groups. As a solution, I suggest that end-of-life policies be structured in ways that emphasize common ground between healthcare providers and surrogate decision-makers while allowing the dissent of surrogate decision-makers to be a genuinely viable option whenever possible.

## Introduction

In "Surrogate Wars: The 'Best Interest Values' Hierarchy & End-of-Life Conflicts with Surrogate Decision-Makers"(henceforth “Surrogate Wars”) Autumn Fiester (2025) argues that incommensurate value systems are often at the heart of end-of-life conflicts between healthcare providers and surrogate decision-makers. Fiester goes on to suggest that the only way to stop the ensuing surrogate wars is for institutions that support the best interest value system to engage more constructively with the surrogate decision-makers who hold the competing value system. While the account of the conflicting worldviews is compelling, there’s a question as to whether these value systems truly are incommensurable and what constructive engagement in end-of-life conflicts should look like. In what follows, I draw on literature on deep disagreement and shared decision-making in health care to make two points. First, I argue that if meaningful engagement is possible in these cases, it is due to the commensurability of these value systems. Second, I argue that to the extent that this meaningful engagement will continue to be difficult to achieve, it is largely due to the hegemony of the best interest value system. Consequently, I call for a framework for end-of-life decision-making that focuses on finding common ground through incompletely theorized agreements while protecting the right for surrogate decision-makers to dissent.

## Competing Value Systems

Fiester argues that end-of-life conflicts are largely the result of clashing value systems. On one side of the conflict, healthcare providers base their decisions on what Fiester refers to as a Best Interest Value system (BIV), which operates on the assumption that life sustaining technologies should only be used for individuals with the prospect of long-term survival in a conscious state. On the other side of the conflict, surrogate decision-makers base their decisions on what Fiester refers to as the Life-Continuation Value system (LCV), which operates on the assumption that, among other things, life sustaining technologies should be used to preserve life whenever possible. Fiester’s objective is to show that regardless of whether BIV is correct, it is a value system in the same way that LCV is a value system. As a result, decision-making that relies on BIV is no more objective than decision-making that relies on LCV.

This is an important insight because it challenges a primary justification for deferring to healthcare providers in end-of-life cases, which is the claim to objective expertise. If the question of what to do in end-of-life cases was primarily a matter of our medical, scientific, and technological possibilities and probabilities, it would be reasonable to defer to the expertise of healthcare providers to determine the best course of action. But as Robert Baker (2019) and others suggest, advancements in our scientific and technological capabilities often create emerging normative questions that go beyond the question of what’s possible. In other words, the sciences and their technologies help us identify the limits of possibility, but whether and how to use technologies can’t be determined by simply focusing on those possibilities. Additionally, as philosophers like Thomas Kuhn (1977) and sociologists like Peter L. Berger and Thomas Luckman (2011) have argued, scientific communities and their norms are subject to the same kinds of value-laden influences that determine broader societal norms. If that is the case in scientific communities more broadly, it makes sense that the reliance on BIV by healthcare providers places them on the same normative terrain as the surrogate decision-makers with whom they are likely to conflict. This equalization of the normative terrain neutralizes the privilege of expertise.

In light of this insight, two claims need to be examined. One claim is that these competing value systems are incommensurable, and the other claim is that surrogate wars can be stopped through constructive engagement. If both BIV and LCV are value systems, that creates the possibility for commensurability that needs further interrogation before we can claim that they aren’t commensurable. The fact that BIV and LCV are both value systems means there is potential for mutual intelligibility, even if each presents radically different ways of understanding the world. For example, despite containing different scripts, grammar, and conceptual apparatuses, the fact that Mandarin and English are both languages means translation and meaningful engagement can happen between the two languages, even if these processes remain incomplete.^1^ Similarly, the fact that BIV and LCV are value systems suggests a strong possibility of engagement between the two systems. At the same time, if the objective is to encourage constructive engagement, it’s important to determine how the relationship between these two value systems could aid or hinder that engagement.

## Deep Disagreement and Incommensurability

If healthcare providers and surrogate decision-makers rely on value-systems that are somehow incommensurable, then one possibility is that both groups are engaged in what some philosophers call deep disagreement (e.g., Aikin & Talisse, 2019 and Ranalli and Lagewaard 2022). In deep disagreement, interlocutors are unable to resolve conflicts because the epistemic reasons that each party offers the other are ineffective due to a lack of common ground and systemically and persistently incompatible commitments. Scott Aikin and Robert Talisse (2019, p. 55) provide the following illustrative example:

[W]hen one party asserts “Birds fly,” and the other says “Birds don’t fly,” they apparently disagree. But if it is discovered that the two parties do not share in common a broad conception of what it is to fly, what things are birds, what authorities to consult, or whether one of them really did see a seagull up in the air just the other day, we should conclude that there is no disagreement after all, but rather a case of mutual unintelligibility.

A real-life example of this type of mutual unintelligibility might be the “false friends” problem between German and English where words like “hose” are spelled and pronounced similarly but have divergent meanings in the two languages.^2^ Something similar might be happening in end-of-life conflicts. Both healthcare providers and surrogate decision-makers might use concepts like “natural death,” “living,” and “futile,” but it’s not at all clear that they are discussing the same things, especially without doing some serious work to reconfigure the underlying value systems that lead to drastically different views of what each of these concepts mean.

 Fiester alludes to the possibility of mutual unintelligibility in discussingdifferences in interpretating concepts like “natural death” or “killing” between the two systems. Given the BIV commitment to only using life sustaining technology when there’s the prospect of long-term conscious survivability, “natural death” likely means something like the removal of those technologies. On the other hand, the LCV commitment to continuing the use of these technologies until physiological functions stop precludes this removal. Both value systems might appeal to the idea of a “natural death,” but it’s likely to mean very different things within each value system. A similar challenge happens when surrogate decision-makers view certain actions as “killing” while healthcare providers view them as clinical activities like “comfort care.”

However, despite the very divergent meanings that adherence to BIV and LCV might attach to concepts like “natural death,” conflict between the two groups might not count as genuine instances of deep disagreement, in which case the two value systems might be fairly commensurable. According to Chris Ranalli and Thirza Lagewaard (2022, p. 6), deep disagreement results from the lack of an adequately shared background. On this view, in order for the end-of-life conflicts between healthcare providers and surrogate decision-makers to be instances of deep disagreement, they also have to result from a lack of sufficiently common ground. If healthcare providers and surrogate decision-makers share enough common ground, then however serious it might be, their disagreement might not actually be deep.

In this case, the problem might be more akin to a dispute about whether Michael Jordan or Lebron James is the greatest basketball player of all time. Let’s call this the G.O.A.T. debate. The disputants in this debate share common ground about the game of basketball and (for the most part) don’t dispute things like the statistics of the game. Rather, the G.O.A.T. debate boils down to the importance disputants place on different elements of that common ground and preferences about the features disputants think ought to be displayed in someone with a claim to the G.O.A.T. title. Ranalli and Lagewaard discuss a related type of disagreement which they call “peer disagreement.” In peer disagreement, a discrepant response from an epistemic peer over something each takes the other to be equally qualified to answer provides reasons for reconsidering one’s own conclusions even if those conclusions aren’t revised.^3^

 Healthcare providers and surrogate decisionmakers are likely not epistemic peers given the medical expertise of the providers. However, both groups might be something like doxastic peers on matters that are ultimately value-dependent, since those matters are in the domain of beliefs and not the technical questions on which healthcare providers are experts. Furthermore, if the BIV and LCV value-systems can be shown to have significant overlap in terms of their higher order or general values, then these value-systems might have enough common ground for end-of-life conflicts to be instances of serious, but not deep, disagreement. A look at these general values suggests that these end-of-life conflicts are more like the G.O.A.T. debate than instances of deep disagreement or, similarly, incommensurability.

For example, while it’s clear that healthcare providers and surrogate decision-makers often mean different things when invoking concepts like “natural death,” “living,” “killing,” and “futile,” each group nonetheless appeals to many of the same concepts. Unlike the aforementioned discrepancy between the English usage of “hose” and the German usage of “Hose,” the disagreement between healthcare providers and surrogate decisionmakers suggests a second-order and not first-order disagreement. In other words, the English and German speakers don’t mean to pick out the same things when they use the same term, so once they clarify their linguistic and lexical context, the discrepancy is easily resolved with each speaker continuing to use the term as they please. The disagreement between healthcare providers and surrogate decision-makers is different since they intend to pick out something like “natural death” as valuable, but disagree about the types of death that count as “natural.” Both groups appeal to the same concept but disagree on the conceptualization. In some ways, this type of disagreement is more difficult to resolve since each party takes themselves to be providing the correct conceptualization of a shared value despite those conceptualizations clashing. Nonetheless, the appeal to the same concept suggests the possibility of finding common ground.

Moreover, surrogate decision-makers could contend that life-continuation values are in the best interest of the patient. In most cases, surrogates likely wouldn’t fight for life-continuation technologies if they genuinely thought it wasn’t in the best interest of the patients, an ostensibly BIV value. The fact that they disagree with healthcare providers on the use of those technologies shows only that they disagree about how to understand the patient’s best interests, not that they don’t consider that a value.On the other hand, healthcare practitioners could contend that they support continuing life sustaining treatment under many circumstances, an ostensibly LCV value. The fact that they disagree with surrogates about life-continuation in marginal cases doesn’t mean they don’t consider life continuation a value.

This way of reframing the two positions suggests that the values of the two groups could be fairly commensurable. Healthcare providers and surrogate decision-makers could still disagree about the best interest of the patient and the appropriate conditions for continuing life sustaining treatment. However, that disagreement could result if one group thinks the other is simply wrong in a particular case, there is difficulty determining the lexical priority of shared values, or there are general, pragmatic challenges of weighing competing considerations. In these cases, conflict would not necessarily be the result of the two groups offering reasons that are mutually unintelligible.

## Incompletely Theorized Agreements in Surrogate Wars

If end-of-life conflicts between healthcare providers and surrogate decision-makers are more like the G.O.A.T. debate than the false friend problem found with languages, then the relevant value systems might not be so incommensurable. That is, if end-of-life conflicts are disagreements between two doxastic peers who can’t agree about low-level particulars, there’s still an opportunity for healthcare providers and surrogate decision-makers to find common ground on higher-level principles and agree about a majority of cases. Afterall, most of the conflict occurs on low-level particulars and marginal cases. This is good news for projects like “Surrogate Wars” that aim at constructive engagement between healthcare providers and surrogate decision-makers since the relative doxastic parity and shared commitments between the two parties make genuine agreement or compromise possible. This agreement and compromise might be possible through what Jennifer Prah Ruger (2009) calls incompletely theorized agreements.^4^ With incompletely theorized agreements, interlocutors find ways to proceed with certain decisions while continuing to adhere to competing worldviews. According to Prah Ruger (2006, p. 19),

An incompletely theorized agreement is one that is not uniformly theorized at all levels, from high level justifications to low level particulars. Incompletely theorized agreements fail to produce depth (full accounts of foundations) or width (coherence with other dimensions), an idea that relates somewhat to John Rawls’s notion of overlapping consensus.

In the case of surrogate wars, there is likely adequate agreement about high level principles such as “act in the best interest of the patient” while there’s a lack of adequate agreement about what the lower-level particulars of that principle entail. As suggested earlier, the fact that healthcare providers and surrogate decision-makers appeal to some of the same concepts suggests that partially theorized agreement might be possible in end-of-life conflicts despite different interpretations of what those concepts mean. For example, such agreement might focus on goals like the alleviation of suffering or the joint objective of respecting the patient’s preferences.

## Hegemony and Power in the Surrogate Wars

 Resolving the nature of the conflict in surrogate wars matters if the recommendation to engage meaningfully or constructively with surrogate decision-makers is to be taken seriously. If this conflict is indeed the result of value systems that are so incommensurable that they constitute deep disagreement, then it’s not clear what meaningful engagement will look like. If this conflict constitutes deep disagreement, then not only are the two parties doomed to continue talking past each other, but the impossibility of mutual intelligibility likely means that resolution of the conflict is likely only possible through the exercise of power. This exercise of power favors healthcare providers given their privileged position within healthcare. The suggestion to incorporate web-based tools to elicit the values and priorities of surrogate decision-makers and patients is helpful, as is the recommendation to examine futility policies like the one at Boston Children’s Hospital that attempt to meaningfully engage with surrogate decision-makers (Fiester, 2025). Nonetheless, it’s not clear that these approaches can genuinely facilitate the engagement between healthcare providers and surrogate decision-makers if members of each group are operating on value systems that are truly incommensurable.

 However, even if these value systems are commensurable, surrogate decision-makers appear to be subordinated when it comes to the final determination. For example, both the Boston Children’s policy and the Texas Advanced Directive Act (TADA) ultimately allow for the unilateral withdrawal of life sustaining treatment over the wishes of surrogate decision-makers.^5^ This provision suggests that the hegemony of BIV remains a concern since its values are likely to prevail whenever values or preferences of healthcare providers and surrogate decision-makers conflict. Consequently, it’s an important empirical question whether the lack of unilateral withdrawal in the types of cases Robert Truog (2012) discusses is the result of a truly egalitarian process were the involved surrogate decision-makers felt comfortable with the results and not merely the instantiation of BIV hegemony. The worry is heightened if, as Fiester compellingly suggests, BIV hegemony is undergirded by utilitarian commitments that skew ableist and ageist. It’s not clear that decisions that are based on those commitments are going to be justifiable to surrogate decision-makers if their value system is so incommensurable with that of healthcare providers. But, if such unilateral withdrawal is justifiable on grounds that don’t have to be acceptable to surrogate decision-makers, then that justification likely stems from considerations that lie outside of the competing value systems , like pragmatic concerns about the stewardship of scarce resources.

 To be clear, I’m not excluding the possibility that unilateral decision-making might be warranted in some of these cases. However, I want to emphasize that this possibility seems to only be available to healthcare providers and not surrogate decisionmakers. While there could be pragmatic reasons for this disparity, the fact that healthcare providers can avail themselves of opportunities for unilateral decisions that are not available to surrogate decision-makers creates the potential for their domination. As philosophers like M. Victoria Costa (2013, p. 923) have argued in discussing societal arrangements like the law of coverture, the fact that some women were lucky enough to have loving and non-interfering husbands did not negate the fact that women were nonetheless dominated by their husbands in those societies. Even the lucky women experienced non-interference only at the mercy of their husbands, a mercy that could be revoked at any moment either through a sudden change of attitude, death, or some other misfortune. Similarly, the fact that there are few if any documented cases of unilateral withdrawal at Boston Children’s or with TADA doesn’t erase the fact that unilateral withdrawal is possible at any moment and would be sanctioned by law and policy.

## Meaningful and Constructive Engagement and the Accommodation of Dissent

 Given this problem, my view is that engagement with surrogate decision-makers can be meaningful and constructive if both parties feel as though there is a genuine possibility of their values being reflected in the conversation, regardless of whether they ultimately get their way. As Cass Sunstein (2016) suggests, engagement between parties with serious disagreement about either high or low-level commitments shouldn’t focus exclusively on finding points of agreement. Instead, the pursuit of incompletely theorized agreements can also be beneficial for understanding disagreement as an important mechanism for highlighting and exploring error and injustice. Afterall, one can reasonably interpret surrogate decision-maker aversion to BIV as motivated by alarm over what they consider deeply morally problematic, unfair, and unjust end-of-life decision-making by healthcare providers.^6^ As a result, meaningful and constructive engagement with surrogate decision-makers requires the facilitation of both agreement and continued disagreement.

 Since this question of what to do in light of continued disagreement cuts across various rationales for BIV, it might be a more fundamental question in the surrogate wars than the question of meaningful engagement more broadly. Regardless of whether BIV and LCV value systems are commensurable, the question of what to do with dissenting surrogate decisions still remains and it seems unlikely that healthcare providers would ever be willing to concede final decision-making authority in the face of continued disagreement. To put it differently, yes, the BIV value-system is hegemonic in contemporary Western-style health care, but the recalcitrant prioritization of the decisions of healthcare providers is as big of a problem as the content of their underlying belief system.

 Fiester seems to acknowledge the problem of hegemony but is insufficiently attentive to the ways in which hegemony impedes meaningful engagement with surrogate decision-makers. To reiterate, the problem is two-fold. First, the BIV value system is privileged healthcare decision-making at the expense of the LCV value system. Second, healthcare providers are privileged as decision-makers at the expense of surrogate decision-makers. The result is that surrogate decision-makers are subordinated while their value system is dismissed, making the prospects of genuine constructive and meaningful decision-making unlikely. The solution would be to make surrogate dissent a viable option that can’t be unilaterally overruled.

## Conclusion

 If engagement with surrogate decision-makers is meant to involve more than getting surrogate decision-makers to agree or simply bypassing them when there’s disagreement, then what’s needed is a framework for preventing healthcare providers from unjustifiably dominating surrogate decision-makers. The point shouldn’t be to merely stop surrogate wars, but also provide a framework through which such stoppage is acceptable to all. This framework should emphasize common ground on high-level principles and commitments while leaving room for disagreement on particular conceptualizations. At the same time, this framework should make the limits of surrogate decision-making very clear. If there are provisions for overriding a surrogate decision-maker’s wishes, good faith engagement requires clearly articulating and disclosing those provisions. Without this level of disclosure and accommodation for at least some of their commitments and principles, it’s hard to believe that surrogates would consider engagement with healthcare providers relevantly meaningful to them. Fully articulating this framework is beyond the scope of this commentary but it is well within the bounds of relevant policies going forward.

## Footnotes

1 For a discussion of the work of conceptual translation between English and Chinese see, e.g., Tai (2005).

2 This phenomenon is found between several language. In this case, the English word primarily means a tubular device (as in a water hose), where “Hose” means pants in German. See, e.g., Domínguez and Nerlichb (2002) for a more expansive account.

3 See Ranalli and Lagewaard (2022, Sect. 3.3).

4 Applying the work of Cass Sunstein to healthcare, Prah Ruger primarily considers this notion within health policy but acknowledges that it might be useful for other types of health-related decision-making see, e.g., Ruger (2006).

5 See Truog (2012, p. 16) for a discussion of the Boston Children’s policy and Fine(2009) for a discussion of the Texas Advanced Directive Act and their provisions for unilateral withdrawal.

6 See for example, Evelyn Kelly’s assertion that doctors at Houston Methodist Hospital were “killing” her son (CBS News, 2018).
